# Adjunctive hemoperfusion with Resin Hemoadsorption (HA) 330 cartridges improves outcomes in patients sustaining multiple Blunt Trauma: a prospective, quasi-experimental study

**DOI:** 10.1186/s12893-023-02056-w

**Published:** 2023-06-03

**Authors:** Roham Borazjani, Salahaddin Mahmudi-Azer, Mohammad Hossein Taghrir, Reza Homaeifar, Gholamreza Dabiri, Shahram Paydar, Hossein Abdolrahimzadeh Fard

**Affiliations:** 1 Trauma Research Center, Shahid Rajaee (Emtiaz) Trauma Hospital, Shiraz University of Medical Sciences, Shiraz, Iran; 2 Department of Intensive Care Medicine, Trauma Research Center, Shahid Rajaee (Emtiaz) Hospital, Shiraz University of Medical Sciences, Shiraz, Iran

**Keywords:** Damage control strategy, Hemoadsorption, Hemoperfusion, Immune response, Trauma, Multi-organ dysfunction

## Abstract

**Background:**

Multi-organ dysfunction syndrome and multi-organ failure are the leading causes of late death in patients sustaining severe blunt trauma. So far, there is no established protocol to mitigate these sequelae. This study assessed the effect of hemoperfusion using resin-hemoadsorption 330 (HA330) cartridges on mortality and complications such as acute respiratory distress syndrome (ARDS) and systemic inflammatory response syndrome (SIRS) among such patients.

**Methods:**

This quasi-experimental study recruited patients ≥ 15 years of age with blunt trauma, injury severity score (ISS) ≥ 15, or initial clinical presentation consistent with SIRS. They were divided into two groups: the Control group received only conventional acute care, while the case group received adjunctive hemoperfusion. P-values less than 0.05 were statistically significant.

**Results:**

Twenty-five patients were included (Control and Case groups: 13 and 12 patients). The presenting vital signs, demographic and injury-related features (except for thoracic injury severity) were similar (p > 0.05). The Case group experienced significantly more severe thoracic injuries than the Control group (Thoracic AIS, median [IQR]: 3 [2–4] vs. 2 [0–2], p = 0.01). Eleven and twelve patients in the Case group had ARDS and SIRS before the hemoperfusion, respectively, and these complications were decreased considerably after hemoperfusion. Meanwhile, the frequency of ARDS and SIRS did not decrease in the Control group. Hemoperfusion significantly reduced the mortality rate in the Case group compared to the Control group (three vs. nine patients, p = 0.027).

**Conclusions:**

Adjunctive Hemoperfusion using an HA330 cartridge decreases morbidity and improves outcomes in patients suffering from severe blunt trauma.

## Background

Trauma has been known as one of the leading causes of death among patients younger than 45 years of age in the United States, and it has been estimated that worldwide more than five million people lose their life to trauma each year [1, 2]. Trauma mortality was described with a trimodal distribution, with immediate deaths at the scene, early deaths due to hemorrhage, and late deaths mostly from multi-organ dysfunction syndrome (MODS) and multi-organ failure (MOF) [3].

Although improving trauma care has led to a decrease in the incidence of multi-organ failure, it still remains one of the major causes of late death following multi-trauma and accounts for 50–60% of all deaths in such patients [4, 5]. The mortality rate reaches up to 100% in patients with more than three failed organs [6]. The role of the immune system in the development of MODS and MOF is well established. In fact, the immune system acts as a double-edged sword; while the system activation results in hemostasis and maintenance of circulatory consistency, the physiological disarray, and over-activation of the immune responses might lead to MODS.

Several factors are associated with the development of MODS-MOF, including trauma severity, hemorrhagic shock, advanced age, massive transfusion, cytokine overproduction, and thrombocytopenia in hospital arrival [7, 8].

Cytokine storm is a hallmark of the immune system overactivation and is highlighted by the overproduction of inflammatory cytokines. The level of pro-inflammatory cytokines and chemokines correlate with trauma prognosis and its potential complications. Interleukin (IL)-6 plays an important role in the acute immune response and seems to be an accurate prognostic factor in trauma. Following severe trauma, high serum levels of IL-8 and interferon (IFN) -γ correlate with the development of acute respiratory distress syndrome (ARDS) and MODS, respectively [9–11]. On the other hand, the dysregulated production of IL-4 and IL-10 suppresses the immune system and increases susceptibility to secondary infections and sepsis [12, 13].

While several procedures such as blood purification methods have been proposed, no specific treatment has been designed to treat cytokine storm and MODS. In the 1940s, adsorbent-based hemoperfusion showed a promising effect in separating urea toxins from the canine blood [14]. When blood passes through the hemoperfusion cartridges membrane, endotoxins and blood cytokines attach to the highly adsorptive ionic membrane, reducing the plasma concentration of pro-inflammatory mediators and cytokines that are known to underline cytokine storm. This procedure effectively dampens the cytokine storm and controls the consequent tissue and organ damage. Also, the effectiveness of extracorporeal cytokine absorber to decreased toxic cytokine levels in severe sepsis has been demonstrated in different studies. The hemoadsorption (HA) 330 cartridges (Jafron, China) are resin-made polymers with highly biocompatible sorbents. HA 330 cartridges can absorb inflammatory cytokines from 500 D to 60 kD such as IL-1, IL-6, IL-8, and TNF-α and subsequently mitigate the incidence and the severity of MODS and ARDS [15, 16].

In this study, we evaluated the utility of hemoperfusion with resin-directed HA330 cartridge in severely injured adult trauma patients who had developed SIRS in spite of appropriate resuscitation and damage control surgery in the acute phase of trauma. We hypothesized that using this adjunctive treatment reduces mortality rate and improves outcomes in such patients.

## Methods

### Study design and setting

This quasi-experimental, prospective controlled trial involving patients with multiple traumas was conducted at the Shahid Rajaee (Emtiaz) hospital, a central trauma care facility in Shiraz, southern Iran, between May 2018 and May 2019. The quasi-experiment design was chosen since the classical randomized controlled trial was too costly and not feasible [17]. We introduced the adjunctive hemoperfusion method to the recruited patients or their substitute decision makers, and they decided whether they wanted to receive this adjunctive treatment. The Institutional Review Board and Research Ethics Committee of Shiraz University of Medical Sciences approved the study with the ethics number: IR.SUMS.MED.REC.1398.505.

### Patient selection

We screened all patients older than 15 years of age who sustained multiple major trauma. All hemodynamically unstable patients had undergone necessary medical and surgical resuscitation measures, including; intravascular fluid administration, vasopressor medications, airway management, coagulopathies correction, and damage control surgery according to the advanced trauma life supports (ATLS) protocol. Survivors were screened 24 h after hospitalization to complete patient enrollment process. Among the resuscitated patients, those with ISS ≥ 15 or initial signs of Systemic Inflammatory Response Syndrome (SIRS) were eligible for enrolment in our study. SIRS defined as the presence of at least two of the followings: (1) body temperature greater than 38.0 °C or less than 36.0 °C, (2) tachycardia (heart rate greater than 90 beats/minute), (3) tachypnea (respiratory rate greater than 20 breaths/minute), (4) leukocytosis (peripheral blood white blood cell count greater than 12 × 10^9^/l) or leukopenia (peripheral blood white blood cell count less than 4 × 10^9^/l) [18, 19]. Patients with any of the following criteria were excluded: intracranial hemorrhage more than 8 mm in size [20], any contraindication for anticoagulation use, including patients with solid organ damage who are candidates for non-operative management [21], damage control surgery, spinal injuries with neurologic deficits, moderate- to high-risk traumatic brain injury and active bleeding [20, 22], and long-term history of immunosuppressive drugs (steroids, cyclosporin, etc.) consumption.

### Intervention

The Control group received conventional care in the intensive care unit (ICU), including appropriate airway management and ventilatory support, appropriate intravascular blood products and vasopressor administration, antibiotic therapy if indicated, correction of coagulopathies, etc. The Case group received conventional care and hemoperfusion via a central venous catheter within the first 6 h of hospitalization. No anti-inflammatory medications were used in either group. A hemodialysis machine (Fresenius Medical Care, Bad Homburg, Germany) was used with an HA330 cartridge for hemoperfusion. A blood flow rate of 100–200 mL/min was set. Hemoperfusion tubes and filters were washed using diluted heparin, and no additional heparin was administered during the hemoperfusion. The hemoperfusion lasted for three hours once a day for three consecutive days.

### Data collection

Demographic data were collected through the admission sheet. Before and after the intervention, the following data were gathered: systolic blood pressure (SBP), diastolic blood pressure (DBP), mean arterial pressure (MAP), heart rate, respiratory rate, shock index (SI), PO2/FIO2 ratio, and Neutrophil to Lymphocyte Ratio (NLR). The data collection before the intervention took place within the 24 h of ED admission for both groups. Data collection after the intervention was set as the last section of hemoperfusion for the Case group completed and the last day of admission for the Control group. The total number of surgical procedures and used packed red blood cells, Hospital Length of Stay (HLOS), and Intensive Care Unit Length of Stay (ICU-LOS) were also reported.

Our primary outcome was the mortality rate between the intervention and the Control group; moreover, the frequency of SIRS and ARDS and their severity before and after the intervention were assessed as secondary outcomes.

### Statistical analysis

Statistical analyses were performed using SPSS software for Windows, version 16.0 (SPSS Inc., Chicago, Ill., USA). There was no missing data; therefore, no statistical imputation was performed. The Kolmogorov–Smirnov test was utilized to evaluate whether the data were normally distributed. Normally distributed data were reported as mean and standard deviation (SD) and compared using the independent t-test, while for non-normally distributed data, median and inter-quartile range (IQR) were reported, and Mann–Whitney U test was utilized to compare them. Categorical variables were reported as numbers and percentages, and the Chi-square test was computed for the association. All statistical tests with two-sided *P*-values of less than 0.05 were significant.

## Results

### Patients’ characteristics

We enrolled twenty-five patients in the study, of whom 12 were in the Case. The study power was 70% according to the primary outcome (mortality rate); therefore, our sample size was enough for this study. Of enrolled patients, only three were female, one in the Case group and two in the Control group. The mean (SD) age for the Case group was 44 (16.5) compared to 42 (7.89) for the Control group. The median (IQR) ISS for the Case group was 21(15–25) and for the Control group was 22 (22–25); however, the difference was not statistically significant (P = 0.09). As shown in Table 1, no significant differences except for thorax AIS and Blood Urea Nitrogen (BUN) were detected between the two groups on hospital arrival. The thorax AIS was significantly higher in the Case group than the Control group (P = 0.01); along with higher BUN in case group (P = 0.02).

**Table 1:** Comparing the demographic and injury-related features between the Case (n = 12) and the Control group (n = 13)

	Case (n = 12)	Control (n = 13)	P-value
Age (Mean, SD)	44(16.5)	42(7.89)	0.85
ISS (Median, IQR)	21(15–25)	22(22–25)	0.09
AIS, median (IQR)			
Head	0 (0–0)^a^	0 (0–0)	1
Face	0 (0–1)	0 (0–0)^b^	0.81
Thorax	3 (2–4)	2 (0–2)	**0.01**
Abdomen	0 (0–3)	0 (0–0)^c^	0.503
Extremities	2 (1–3)	0 (0–3)	0.18
External	0 (0–1)	0 (0–3)	0.35
Male gender, N (%)	11(91.6%)	11(84.6%)	1
Mechanism of injuries, N (%)			
RTA	10(83.3%)	10(76.9%)	0.61
Assault	0(0%)	1(7.7%)	
Falling	2(16.7%)	2(15.4%)	
WBC (Mean, SD)	14,367 (6894)	14,908 (6476)	0.84
Hb (Median, IQR)	10.85 (9.40–13.60)	13.30 (10.60-14.05)	0.19
Plt (Mean, SD)	227,583 (94,048)	193,167 (52,477)	0.28
INR (Mean, SD)	1.23 (0.22)	1.16 (0.21)	0.47
PTT (Median, IQR)	32 (30–35)	31 (30–36)	0.86
BUN (Median, IQR)	20 (16.5–26)	16 (13.5–17)	**0.02**
Creatinine (Median, IQR)	1.60 (1.02–2.28)	1.23 (0.85–1.45)	0.14
Blood sugar (Mean, SD)	151 (46)	159 (78)	0.76
Fibrinogen (Mean, SD)	331 (115)	302 (133)	0.60

SD, Standard Deviation; IQR, Inter-Quartile Range; ISS, Injury Severity Score; AIS, Abbreviated Injury Scale, RTA; Road Traffic Accident; WBC, White Blood Cell, Hb; Hemoglobin, Plt; Platelet, INR; International Normalized Ratio, PTT; Partial Thromboplastin Time, BUN; Blood Urea Nitrogen

^a^ Two out of 12 patients had Head AIS = 3,

^b^ Two out of 13 patients had Face AIS = 3,

^c^ Out of 13 patients, one had Abdomen AIS = 4, and two had abdomen AIS = 3

### Measured outcomes

During our study, 12 participants died, of whom three were from the Case group (Table 2). Mortality was significantly lower in the group that received HA330 hemoperfusion (P < 0.05). The Case group received more packed RBC and stayed longer in the ICU (P < 0.05). The decreased mortality rate in the Case group may account for these findings.

The incidence of the ARDS differed significantly in the Case and Control groups. ARDS was defined as the acute onset of respiratory failure, together with bilateral infiltrates on chest radiograph, hypoxemia as defined by a PaO_2_/FiO_2_ ratio ≤ 200 mmHg, and no evidence of increased left atrial pressure. Before starting the trial, eleven patients (91.6%) in the Case group had ARDS, which significantly declined after the intervention (three patients, 25%). Given the high mortality in the Case group the analysis of the ARDS in the Case group over time was not entirely possible, although; during the time of intervention, the number of patients with moderate ARDS in the Control group rose from three to five patients.

**Table 2:** Comparison of outcome measures between the groups (n = 25)

Outcomes	Case (n = 12)	Control (n = 13)	P-value
Mortality rate, N (%)	3(25%)	9(69.2%)	**0.027**
Surgical procedures (Median, IQR)	3 (2–5)	1 (0–2)	**0.002**
Packed RBC (Median, IQR)	7 (4–13)	2 (0–8)	**0.046**
Survivors’ HLOS (Mean, SD)*	36.67 (16.66)	18.75 (18.25)	0.109
Survivors’ ICU-LOS (Mean, SD)*	28 (16.80)	9 (4.83)	**0.011**

SD, Standard Deviation; RBC, Red Blood Cell; ICU, Intensive Care Unit; IQR, Inter-Quartile Range; HLOS, Hospital Length of Stay; ICU-LOS, Intensive Care Unit Length of Stay

*the mean of HLOS and ICU-LOS was calculated among survivors of the Case (9 patients) and the Control group (4 patients)

All of the patients in the Case group had signs of SIRS before the intervention, which dropped significantly to 25% (three patients) after the intervention. For the Control group, signs of SIRS were detected in four individuals (30.8%), which increased by 15.4% after the conventional intervention.

Table 3 outlines the clinical variables before and after intervention between the Control and the Case groups. All patients in the Case and Control groups received vasopressor. Among all variables investigated in this study, only PO2/FIO2 ratio was significantly increased in participants of the Case group after performing resin hemoperfusion.

**Table 3:** Comparison of clinical variables before and after intervention between groups

Variables	Group	Before intervention	After intervention	Delta*	P-value
**SBP** (Mean, SD)	**Control**	110.84 (17.52)	111.84 (24.19)	1 (28.85)	0.36
	**Case**	114.83(11.07)	124.58 (17.69)	9.75 (16.57)	
**DBP** (Mean, SD)	**Control**	67.84 (14.77)	66.07 (9.97)	-1.77 (14.69)	0.45
	**Case**	70.75 (6.35)	74.16 (17.98)	3.41 (19.32)	
**HR** (Median, IQR)	**Control**	82 (75–112)	90 (75–100)	2 (-6–9)	0.82
	**Case**	99 (75 − 11)	89 (79–109)	1(-19–14.5)	
**RR** (Median, IQR)	**Control**	20 (16–20)	16 (14–20)	0 (-5–4)	0.54
	**Case**	18 (17–21)	19 (16–21)	-2 (-2–0.5)	
**SI** (Mean, SD)	**Control**	0.88 (0.48)	0.80 (0.17)	-0.08 (0.44)	0.96
	**Case**	0.83 (0.20)	0.76 (0.26)	-0.07 (0.22)	
**MAP** (Mean, SD)	**Control**	82.17 (14.46)	81.33 (12.95)	-0.84 (15.89)	0.35
	**Case**	85.44 (7.27)	90.97 (17.16)	5.53 (17.65)	
**PaO2/FIO2** (Mean, SD)	**Control**	304.90 (137.97)	300.57 (143.84)	-4.33 (217.60)	0.019 ^a^
	**Case**	198.45 (111.79)	394.50 (149.67)	196.04 (173.27)	
**NLR** (Median, IQR)	**Control**	5.64 (3.72–11.99)	4.90 (4.20–6.10)	-1.28 (-5.18–0.48)	0.88
	**Case**	11.16 (6.24–17.67)	7.39 (3.23–15.48)	-3.64 (-6.28–2.56)	

SBP, Systolic Blood Pressure; DBP, Diastolic Blood Pressure; HR, Heart Rate; RR, Respiratory Rate; SI, Shock Index; MAP; Mean Arterial Pressure; NLR, neutrophil to Lymphocyte Ration; SD, Standard Deviation; IQR, Inter-Quartile Range; NLR, Neutrophil/Lymphocyte Ratio

* The changes between the values of before and after intervention

^a^ Statistically Significant

## Discussion

Treatment of critically ill trauma patients poses a global challenge. This challenge has led to the studies seeking novel treatment methods. Indeed, traumatic brain injury, hemorrhagic shock, and physiological sequelae (acidosis, hypothermia, coagulopathy) are the most common causes of early death and correlate with the severity of the index injury [23–25].

However, the most common causes of delayed mortality in trauma patients are multi-organ failure and infection, which are underlined by complications of the impaired immune response [26, 27]. The severity of immune dysregulation correlates with the severity of the trauma, and this response begins in the early hours after injury and lasts for several days [28]. Hence, the level of inflammatory cytokines in the golden hours of trauma has an important role in delayed phase mortality and morbidity [12, 29, 30].

With increasing awareness about the role of immune response in severe trauma, the concept of damage control has been recommended in the resuscitation and operation of critically injured patients. This concept reduces immune dysregulation and leads to faster correction of consequent physiological disorders [31].

Although many studies have shown that damage control surgery and resuscitation reduce the early mortality among trauma patients, MODS contributes to late phase mortality. Therefore, blood purification methods and damage control management may improve the prognosis of critically injured trauma patients presenting with signs and symptoms of cytokines storm syndrome. However, no definite standard care for such patients has been introduced yet [32, 33].

Several blood purification methods were assigned to mitigate the inflammatory responses. Almost all of them were conducted in non-trauma settings. A form of Continues Renal Replacement Therapy technology known as Continues Veno-Venous Hemofiltration (CVVH) has been used to manage renal failure, ARDS, cardiogenic pulmonary edema, and sepsis. CVVHs eliminate small to medium-sized inflammatory mediators critical in the pathophysiology of SIRS.

We believed that our study was among the first to assess the efficacy of the blood purification method among trauma patients. We used HA 330 filter since previous studies confirmed its modulatory effects. **Laping Chu et al.** compared the effect of hemofiltration with HA330 and pulse high-volume hemofiltration (HF-PHVHF) with CVVH among patients with septic shock. They have shown that HF-PHVHF was superior to the CVVH since HF-PHVHF resulted in more reduction in serum levels of IL-6, IL-10, and TNF-α. The mortality rate among patients who received HF-PHVHF was lower than the CVVH group (26.7% vs. 40%) [34]. **He Z et al.**. concluded that using hemoadsorption reduces the inflammatory responses and facilitates recovery among patients with cardiopulmonary bypass [35]. Even amid the COVID-19 pandemic, several studies used the HA330 filter to improve patients’ outcomes [36, 37].

Immune dysregulation plays a pivotal role in the pathophysiology of late-onset trauma complications. Therefore, we hypothesized that early reduction in the inflammatory cytokines from the peripheral blood (as an adjunct to damage control strategy) might decrease the late-onset complications and improve outcomes.

In the recent study, all patients in the Case group and four patients in the Control group had signs and symptoms of SIRS on ICU arrival. As the SIRS index has been known as a predictor of mortality for trauma patients, one would have expected that mortality should have been higher in the Case group. However, our findings showed that mitigating inflammatory response in the initial hours following trauma (the golden hour) can improve outcomes. Moreover, hemoperfusion resulted in the resolution of the signs and symptoms of SIRS in the majority of the patients in the Case group. Meanwhile, the SIRS frequency was increased in the Control group (four vs. six patients before and after the conventional treatment). Finally, the Case group had a higher rate of ARDS at the beginning of ICU admission and before the intervention. But, at the end of the experiment, three and eight patients in the Case and the Control groups suffered from ARDS, respectively. Several factors are attributed to the ARDS development. The severity of thoracic injuries, the amount of fluid received during resuscitation, and the immune-pathogenesis mechanisms [38]. Although the Case group had higher Thorax AIS, we believed that our purification method mitigated the two other factors leading to a lower rate of ARDS development.

Despite the shortcomings of our study, including the limited number of patients and our inability to measure the serum level of the inflammatory cytokines, our study showed some promising data regarding the utility of hemoperfusion in the management of severe trauma. Another limitation was that we did not monitor the dynamic cardiovascular indices such as cardiac output, cardiac index, and systemic vascular resistance. One of the potential disadvantages of the quasi-experimental design is that the intervention may not be well established. To avoid this pitfall, two senior researchers (H. AF. And R.B.) separately monitored each intervention. We hope other studies could further examine this concept with a larger number of patients enrolled and more detailed information about the level of inflammatory markers before and after hemoperfusion.

## Conclusion

The immune-modulatory effect of hemoperfusion may significantly decrease the mortality rate in patients with severe trauma within golden hours following injuries. Moreover, the frequency of ARDS and SIRS were significantly reduced when comparing the Case and the Control groups, potentially by mitigating the dysregulated immune response.

## Data Availability

The datasets used and/or analyzed during the current study are available from the corresponding author on reasonable request.

## Abbreviations

HA330: Hemoadsorption 330
ARDS: Acute Respiratory Distress Syndrome
SIRS: Systemic Inflammatory Response Syndrome
ISS: Injury Severity Score
MODS: Multi-Organ Dysfunction Syndrome
MOF: Multi-Organ Failure
IL: Interleukin
IFN: Interferon
ATLS: Advanced Trauma Life Supports
SIRS: Systemic Inflammatory Response Syndrome
ICU: Intensive Care Unit
SBP: Systolic Blood Pressure
DBP: Diastolic Blood Pressure
MAP: Mean Arterial Pressure
SI: Shock Index
HLOS: Hospital Length of Stay
ICU-LOS: Intensive Care Unit Length of Stay
NLR: Neutrophil to Lymphocyte Ratio
CVVH: Continues Veno-Venous Hemofiltration
HF-PHVHF: Hemofiltration with HA330 and Pulse High-Volume Hemofiltration
